# Pattern of antimicrobial prescription in Africa: a systematic review of point prevalence surveys

**DOI:** 10.11604/pamj.2023.45.67.36191

**Published:** 2023-05-29

**Authors:** Ijeoma Nkem Okedo-Alex, Ifeyinwa Chizoba Akamike, Ihoghosa Iyamu, Chukwuma David Umeokonkwo

**Affiliations:** 1 Department of Community Medicine, Alex Ekwueme Federal University Teaching Hospital, Abakaliki Ebonyi State, Abakaliki, Nigeria; 2 African Institute for Health Policy and Health Systems, Ebonyi State University (EBSU), Abakaliki, Nigeria; 3 School of Population and Public Health (SPPH), University of British Columbia, Vancouver, Canada,; 4 Pan-African Research Consortium, Federal Capital Territory (FCT), Abuja, Nigeria

**Keywords:** Antibiotics, antimicrobial, prescription, point prevalence, survey, Africa, systematic review

## Abstract

**Introduction:**

inappropriate use of antimicrobials is a cause for concern and contributes to the global antimicrobial resistance crises especially in Africa. This review aims to summarize the available evidence on the point prevalence and pattern of antimicrobial and/or antibiotic prescription in Africa.

**Methods:**

this review was carried out between April and September 2021 and identified published studies up until March 2021 on the point prevalence of antibiotic and/or antimicrobial use in Africa. Sources searched were OVID, PubMed, EMBASE, CINAHL, Web of Science, Google Scholar, Google, and African Journal Online (AJOL). Observational studies that reported prevalence published in English language were included. Covidence systematic review software was used for this review. A form for data extraction using domains culled from the Global Point Prevalence Survey of Antimicrobial Consumption and Resistance (Global-PPS) was developed on Covidence. Screening of studies for eligibility was done independently by two reviewers. Critical Appraisal tool for use in Joanna Briggs Institute (JBI) Systematic Reviews for prevalence studies was used for quality appraisal.

**Results:**

a total of 17 studies that met the inclusion criteria were included in the review. The overall prevalence of antimicrobial/antibiotic use among inpatients in these studies ranged from 40.7% to 97.6%. The median antimicrobial/antibiotic use was 61.3 [IQR= 45.5-72.1]. The highest use of antimicrobials was reported among studies from Nigeria with a prevalence of 97.6%. The most prescribed antibiotics were the beta-lactam penicillin (Amoxicillin, clavulanic acid) (86.9%), and third generation cephalosporins (55.0%). There was general preference for parenteral route of administration of the antimicrobial agents (40-70%). Use for community acquired infections (28.0-79.5%) was the main reason for use. Majority of the prophylactic use of antimicrobial agents were for surgical prophylaxis. **Conclusion:** the high prevalence of antimicrobial use in Africa reinforces the need for continued surveillance and concerted efforts to institutionalize and support antimicrobial stewardship for prescribers in health institutions in the African region.

## Introduction

Antimicrobials are important agents in reducing morbidity and mortality among patients who have infections, however, the frequent inappropriate and excessive use of antimicrobials is a cause for concern because of the risk of antimicrobial resistance [1-3]. Factors that contribute to the global AMR crises include overprescribing and over-dispensing of antimicrobial drugs by health workers, patient noncompliance with therapy, poor quality medicines, wrong prescription and dosage, and poor infection prevention and control practices [4]. The highest AMR burden is seen in low and middle-income countries including countries in Africa. A range of activities and programs to enhance appropriate antibiotic prescribing ranging from global, regional and national levels have been carried out as a result of the concerns with the increasing rate of AMR. In response to the AMR crises, the World Health Organization (WHO) developed a Global Action Plan (GAP), with a goal to ensure the successful treatment and prevention of infectious diseases using effective and safe medicines that are quality-assured. These medicines are to be used in a responsible way, and expected to be accessible to all who need them [5]. Point prevalence survey (PPS) is a necessary means of providing accurate data on the current antibiotics utilization [6,7]. It provides information that can be used to improve antimicrobial use in hospitals and thereby reduce resistance [6,7]. Additionally, it can be used to monitor antimicrobial stewardship and infection control programs. The aim of developing the WHO PPS methodology [8] was to collect baseline information on antibiotics use in hospitals. This information is collected from medical records and other associated patient records relevant for treatment and management of infectious diseases and is expected to be repeated once every few years [4]. The specific objectives of the methodology include providing a standardized methodology which can be used to estimate the prevalence of antibiotics use in hospitals and provide information on prescribing patterns; and also make available information for decision making by policy makers [4]. Although, several PPS studies have reported varying prevalence of antibiotics use and provided insight into current antibiotic prescribing practices, there is a dearth of evidence synthesis on this subject in Africa [7,9-12]. This review aims to summarize the available evidence on the point prevalence and pattern of antimicrobial and/or antibiotic prescription in Africa in order to identify areas for improvement and inform future policies.

## Methods

For screening eligible studies, this review utilized the Preferred Reporting Items for Systematic Reviews and Meta-Analyses [PRISMA] checklist for reporting a systematic review or meta-analysis protocol [13].

**Protocol registration**: the protocol can be found at [14] with registration number: CRD42020215879 [14].

**Search strategy**: this review was carried out between April and September 2021 and identified published studies up until March 2021 on the point prevalence of antibiotic and/or antimicrobial use in Africa. The following databases and sources were searched: OVID, PubMed, EMBASE, CINAHL, Web of Science, Google scholar, Google, and African journal online (AJOL). Some of the terms employed in the search include but are not limited to the following: prevalence, point prevalence, antibiotic, antimicrobial, prescription use, Africa. The search strategy is contained in (Annex 1, Annex 2).

**Inclusion criteria**: the eligibility criteria for inclusion of studies include cross-sectional studies regardless of publication period published or retrievable in English language, and studies conducted in Africa. The study must report point prevalence of antimicrobial/antibiotic prescription or use in health care settings using point prevalence survey tools.

**Exclusion criteria**: the studies excluded were those not published in English language, multi-country studies, reviews, editorials, commentaries, case reports, case series and qualitative.

**Figure 1:**
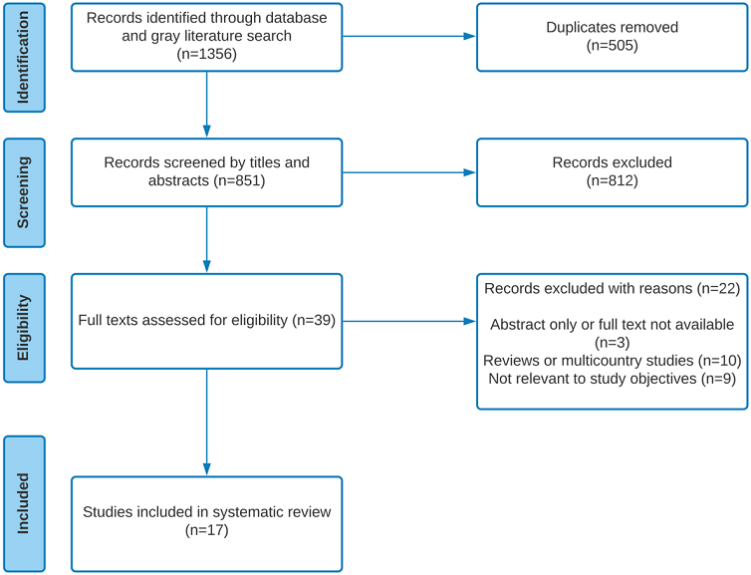
prisma diagram showing the process of selection and inclusion of studies

**Data extraction**: the authors utilized the Covidence systematic review software for this review [15]. Two of the reviewers (INO and ICA) independently screened the titles and abstracts and all the reviewers were involved in data extraction from the full texts. The Covidence software automatically kept track of consensus and conflicts which were resolved by discussions. References of the published studies were managed using the Mendeley reference manager. The reviewers jointly developed a form for data extraction on Covidence using domains culled from the Global Point Prevalence Survey of Antimicrobial Consumption and Resistance (Global-PPS) [16]. Some of the fields in the extraction form were first author´s name, year the study was conducted, year the study was published, study location and setting, country of study, title/objective, study design, sample size, prevalence type (antibiotic/antimicrobial), prevalence of antibiotic/antimicrobial use (for studies involving multiple sites with no reported overall prevalence, the average value was taken as the overall prevalence), prevalence of most used antibiotic, prevalence of least used antibiotic, route and indication for use, stop/review dates etc. The process through which the articles were selected and included is shown in Figure 1.

**Quality appraisal**: all reviewers participated in the quality appraisal process which was done using the Critical Appraisal tools for use in JBI Systematic Reviews tool for prevalence studies [17] (Annex 3). This quality assessment tool contained nine questions which explored the adequacy of the sample frame, sampling of study participants, adequacy of sample size, description of study subjects and setting, data analysis conducted with sufficient coverage of the identified sample, use of valid methods used for the identification of the condition, response rate and appropriate statistical analysis amongst others. A final assessment to include or exclude was also part of this tool. For each variable, the options to score from were Yes, No, Unclear, and not applicable. The adequacy of the sample size was not applicable to any of the studies included in this review as sample size calculation is not required by the PPS tool. For the other parameters, all the seventeen studies that fulfilled the inclusion criteria were scored “Yes” and a final decision was made to include them in the study.

**Patient and public involvement**: it was not appropriate nor possible to involve patients or the public in this work.

## Current status of knowledge

Among 1356 studies returned in our database search, 505 duplicates were identified and removed. Further, 812 studies were excluded during the title and abstract review, resulting in thirty-nine full texts being assessed for eligibility. Of these, nine were removed because the study was not relevant to the study objective, ten removed because there were review articles or they were multi-country studies and three were excluded because they were either only abstract or the full text were not available. A total of 17 studies were included in the review. A total of 17 articles [6,7,9-11,18-29] published between 2017 and 2021 were included in the review. The studies were all cross-sectional design using mostly the global PPS, WHO PPS or ECDC PPS tools (Table 1). Five studies were conducted in Nigeria, four from Ghana, three from Kenya, two from South Africa and Tanzania respectively and one from Benin. The studies were conducted in West, Central and South African region involving over 14,000 patients (Table 1). The overall prevalence of antimicrobial/antibiotic use among inpatients in these studies ranged from 40.7% to 97.6%. Nigeria had the highest prevalence (97.6%) while South Africa had the lowest prevalence of antimicrobial/antibiotic use (37.7%). The median antimicrobial/antibiotic use was 61.3% (interquartile range [IQR] (45.5-72.1). The most prescribed antibiotics were the beta-lactam penicillin (Amoxicillin, clavulanic acid) (86.9%), and third generation cephalosporins (55.0%). The second most-used antimicrobial apart from cephalosporins (44.7%) was nitroimidazoles/ metronidazole (41.8%) (Table 2).

**Table 1:** characteristics of studies included in the review

Author name	Year of Publication	Country	Study design	Sample size	Number of facilities	Tool used
Afriyie [18]	2020	Ghana	Cross-sectional	213*	2	Global PPS
Seni [9]	2020	Tanzania	Cross sectional	948	6	WHO PPS
Umeokonkwo [19]	2019	Nigeria	Cross-sectional	220	1	Global PPS
Maina [20]	2020	Kenya	Cross-sectional	3590	14	Global PPS
Momanyi [10]	2021	Kenya	Cross sectional	179	1	Global PPS
Horumpende [7]	2020	Tanzania	Cross sectional	399	3	ECDC PPS
Okoth [21]	2018	Kenya	Cross-sectional	269	1	Modified PPS
Abubakar [29]	2020	Nigeria	cross-sectional	321	3	ECDS PPS
Bediako-Bowan [22]	2019	Ghana	Cross sectional	540	10	ECDC PPS
Fowotade [23]	2020	Nigeria	Cross-sectional	451	1	Global PPS
Oduyebo [24]	2017	Nigeria	Cross-sectional	828	4	Global PPS
Ahoyo [11]	2014	Benin	Cross sectional	3130	39	Not available
Labi [25]	2019	Ghana	Cross-sectional	2107	10	ECDC PPS
Labi [26]	2018	Ghana	Cross-sectional	677	1	ECDC PPS
Kruger [6]	2021	South Africa	Cross sectional	181	1	ECDC PPS
Nnadozie [27]	2020	Nigeria	Cross-sectional	82	1	Global PPS
Dlamini [28]	2019	South Africa	Cross-sectional	512	1	PPS

* Frequencies extrapolated from percentages presented in paper, PPS: Point prevalence Survey, ECDC: European Centre for Disease Prevention and Control.

**Table 2:** prevalence and pattern of antimicrobial/antibiotic use

Author name	Country	Type of Overall prevalence	General prevalence of use	Most used Antimicrobial	Prevalence of most used Antimicrobial	Second most used Antimicrobial	Prevalence of second most used Antimicrobial
Afriyie 2020 [18]	Ghana	Antibiotic	73.5+	NA	NA	NA	NA
Seni 2020 [9]	Tanzania	Antibiotic	62.3	Ceftriaxone	30.9	Metronidazole	22.9
Umeokonkwo 2019 [19]	Nigeria	Antimicrobial	78.2	Ceftriaxone	25.1	Metronidazole	24.6
Maina 2020 [20]	Kenya	Antimicrobial	47.0^	Cephalosporins	26.0	Nitroimidazole	20.0
Momanyi 2021 [10]	Kenya	Antibiotic	54.7	Penicillin	46.9	Cephalosporins	44.7
Horumpende 2020 [7]	Tanzania	Antibiotic	44.0	Ceftriaxone	29.8	Metronidazole	23.9
Okoth 2018 [21]	Kenya	Antibiotic	67.7	3^rd^ Gen cephalosporins	55.0	Imidazole/Metronidazole	41.8
Abubakar 2020 [29]	Nigeria	Antimicrobial	80.1	Metronidazole	30.5	Ciprofloxacin	17.1
Bediako-Bowan 2019 [22]	Ghana	Antibiotic	70.7	Nitroimidazoles	25.6	2^nd^ & 3^rd^ Gen Cephalosporins	20.0
Fowotade 2020 [23]	Nigeria	Antimicrobial	59.6	3^rd^ Gen cephalosporins	23.9	Metronidazole	18.0
Oduyebo 2017 [24]	Nigeria	Antimicrobial	69.7	Ceftriaxone	18.9	Metronidazole	18.0
Ahoyo 2014 [11]	Benin	Antimicrobial	40.7	Beta-Lactam*	86.9	Cephalosporin**	17.4
Labi 2019 [25]	Ghana	Antibiotic	61.3	-	-	-	-
Labi 2018 [26]	Ghana	Antibiotic	51.4	Metronidazole	17.5	Amoxicillin-clavulanic acid	13.4
Kruger 2021 [6]	South Africa	Antimicrobial	44.0	Amoxicillin and Clavulanic acid	-	Cotrimoxazole	-
Nnadozie 2020 [27]	Nigeria	Antimicrobial	97.6	Metronidazole	32.3	Ceftriaxone	28.4
Dlamini 2019 [28]	South Africa	Antimicrobial	37.7	Broad spectrum penicillin	34.1	Cephalosporin	17.9

* (Ampicillin, cloxacillin, amoxicillin, amoxicillin-acid clavulanic) **(Ceftriaxone, cefotaxime, ceftazidime) +average value ^antibiotic use prevalence separately reported as 94%

**Figure 2:**
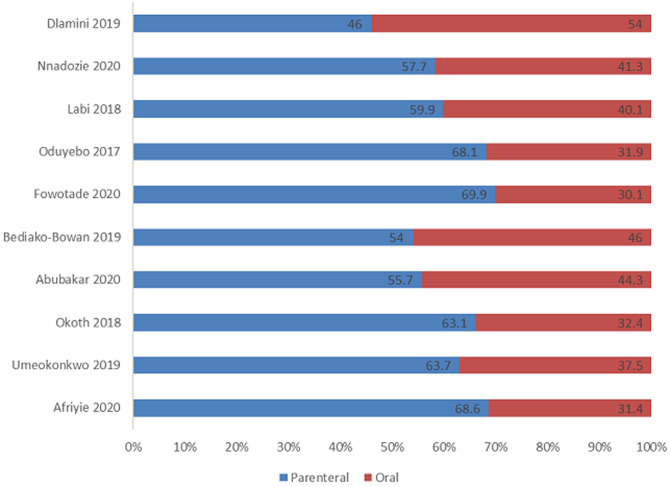
distribution of route of antimicrobial administration among the studies

**Figure 3:**
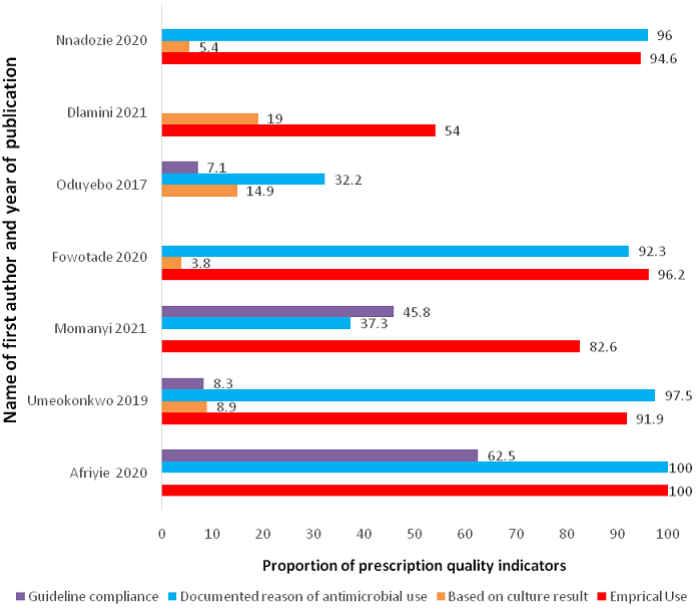
distribution of prescription quality indicators reported among the studies

The majority of the studies (10/17, 58.8%) reported the route of administration of the antimicrobial agent used. There was general preference for parenteral route of administration of the antimicrobial agents (40-70%) (Figure 2). The indication for use of antibiotics was reported in majority of the studies. Regarding the therapeutic indications, most reasons for the use of antibiotics were for community-acquired infections (28.0-79.5'5%). Some studies also reported significant proportion of the prescriptions were for hospital-acquired infections (19.1-30.8%). The majority of the prophylactic use of antimicrobial agents was for surgical prophylaxis (Table 3). However, some significant use for medical prophylaxis were also reported (15;5.1-40.9%). Some studies also reported some therapeutic and prophylactic use of antimicrobial agents for which there was no clear diagnosis. There were variations in reporting prescription quality indicators due to the focus of the studies and the type of tools they employed. Among the studies that reported prescription quality indicators, there was wide variations in the proportion reported. Most studies reported poor utilization of laboratories in making bacteriological diagnosis of infection as most antimicrobial prescription were done in empirical basis. It was also evident that there was little or no guidelines available in most of the studies settings. Where guidelines were available for antibiotic prescription, these were largely not complied with (0%-62.5'5%) (Figure 3).

**Table 3:** indication for use of antimicrobial agents

Author name	Therapeutic Indication		Prophylactic indication		Other indications		Treatment unspecified	
	Community acquired infection (%)	Hospital acquired infection (%)	Surgical (%)	Medical (%)	Others (%)	Unknown (%)	Prophylaxis (%)	Therapeutic (%)
Afriyie 2020 [18]	79.5	20.5	59.1	40.9	-	-	-	-
Seni 2020 [9]	36.7	-	30.2	24.0	-	-	-	-
Umeokonkwo 2019 [19]	45.1	6.0	44.0	2.9	-	1.6	48.6	51.6
Momanyi 2021 [10]	54.2	2.8	29.1	15.1	-	-	-	75.4
Horumpende 2020 [7]	42.0	10.0	30	0.5	-	11.0	-	-
Okoth 2018 [21]	28.0	13.0	22	29	6.0	2.0	-	-
Abubakar 2020 [29]	38.7	16.3	22.5	14.9	-	7.6	-	-
Bediako-Bowan 2019 [22]	77.7	22.3	84.0	16.0	-	-	37.7	58.6
Fowotade 2020 [23]	69.2	30.8	70.1	29.9	-	1.6	56.1	42.3
Oduyebo 2017 [24]	45.8	5.4	27.1	11.7	10	-	-	51.2
Ahoyo 2014 [11]	-	19.1	-	-	-	-	-	-
Labi 2018 [26]	40.1	-	33.6	5.4	-	16	-	-
Nnadozie 2020 [27]	34.1	9.0	56.9	0	-	-	57.0	43.0

In this systematic review of publications assessing the prescription pattern and use of antimicrobials based on the various forms of the PPS methodology in Africa, we found a generally high prevalence of antimicrobial prescription and use. We also found considerable variation in antimicrobial use among studies from various countries, with Nigeria having the highest prevalence of antimicrobial use and Benin and South Africa reporting the lowest prevalence of antimicrobial use. Further, we found that the most prescribed antibiotics were third generation cephalosporins, beta-lactam penicillin and the Nitroimidazoles (including metronidazole). This review also showed that the most common indications for antimicrobials were for community acquired infections and for prophylaxis in surgical and medical cases. Our systematic review provides an in-depth understanding about antimicrobial prescription and use patterns in Africa. First, unlike previous studies which suggested limited use of the PPS methodology in Africa, we found 17 studies using various versions of the methodology, suggesting increasing concerns about tracking antimicrobial use which is a key strategy in the global action plan an antimicrobial stewardship [5]. The high prevalence of antimicrobial use found in our study is similar with other global studies that highlight inappropriate use of antimicrobials in the region as a global health problem [30]. However, the prevalence of antimicrobial use in this study was relatively higher than reported in other regions of the world including Europe and North America [30,31]. Studies have found higher antimicrobial stewardship levels and awareness of antimicrobial use among prescribers in South Africa and this could explain the lower levels of antimicrobial use compared to other countries [32,33].

The variation in antimicrobial prescription may be due to varied existence and adherence to antimicrobial prescription guidelines including a large dependence on empirical basis for prescription, variations in prevalence of infectious diseases (including community- and hospital-acquired infections) and sub-standard infection prevention and control practices in hospitals [30]. Further, our findings of broad-spectrum antibiotics including third generation cephalosporins and beta-lactam penicillin suggests that clinicians may be adopting empirical use of broad-spectrum antibiotics as a strategy to manage infections in jurisdictions where access to diagnostic services including microbial culture and susceptibility testing may be limited [30,34]. It could also reflect rising rates of multi-drug antimicrobial resistance which have been documented as having a significant burden in low- and middle-income countries including those in Africa [34].

The findings from our systematic review are of clinical and global health importance considering the rising threat and burden of antimicrobial resistance in the region. Systematic misuse and overuse of antimicrobials among human populations, especially in-hospital settings continue to contribute significantly to the global health threat of antimicrobial resistance [34,35]. Findings from our systematic review suggest that there is need for sustainable antimicrobial stewardship programs with standardized antimicrobial prescription guidelines within health systems in the region. For optimal adherence, it is also important that there is strengthened capacity-building among clinicians in the region, ensuring that these guidelines and minimum standards that enable good institutional antimicrobial prescription practices are adhered to. Optimal infection prevention and control practices can also motivate correct antimicrobial prescription especially for prophylaxis [35]. However, we must note that challenges regarding care provider-patient interactions only contribute a fraction to the issues, as there are concerns about the quality of antimicrobials that also influence prescription behavior [35].

Findings from this study should be considered in view of its inherent limitations. Firstly, the variety of the tools, varied types of prevalence reported (antimicrobial/antibiotic) as well as different patient populations targeted by the included papers implied that we were unable to make comparisons between the studies and their varied jurisdictions due to the non-equivalence of some of their findings. Further, our study only included articles published in English. Considering that some articles from Africa may have been published in French, this may introduce some bias into this review. However, given the observed trends in the data, we are confident that our review is sufficient to demonstrate the current state of antimicrobial prescription and use on the continent.

## Conclusion

Our study demonstrates the utility of the PPS tools to assess the prevalence and patterns of antimicrobial use within hospitals, providing a basis for quality improvement in line with the WHO GAP on antimicrobial stewardship. The high prevalence of antimicrobial use in sub-Saharan Africa reinforces the need for continued surveillance and concerted efforts to institutionalize and support antimicrobial stewardship for prescribers in health institutions in the region.

### What is known about this topic

- *Antimicrobial resistance is a growing concern globally;*
- *Point prevalence surveys have been useful in describing the use of antimicrobials and health facilities*.

### What this study adds

- *There review showed a high prevalence of antimicrobial prescription across African regions and countries;*
- *Parenteral administration, surgical prophylaxis, empirical basis and antimicrobial use for community-acquired infections are prevalent in African health settings*.

## Annexes

**Annex 1**: PRISMA checklist for reporting a systematic review or meta-analysis protocol (PDF-395 ko)

**Annex 2**: search strategy for the systematic review on the point prevalence and pattern of antimicrobial prescription in Africa (PDF-396 ko)

**Annex 3**: quality appraisal tools for studies included in the systematic review on the point prevalence and pattern of antimicrobial prescription in Africa (PDF-408 ko)
